# Anaplastic Large Cell Lymphoma Occurring in an HIV-Positive Patient

**DOI:** 10.1155/2012/180204

**Published:** 2012-12-12

**Authors:** Philippe Genet, Driss Chaoui, Virginie Masse, Ahmad Al Jijakli, Nina Arakelyan, Laurent Sutton

**Affiliations:** Service d'Hématologie, Centre Hospitalier Victor Dupouy, 69 Rue du Lieutenant-Colonel Prudhon, 95100 Argenteuil, France

## Abstract

Cases of anaplastic large-cell lymphoma (ALCL) of T phenotype are sparse in the setting of HIV patients. We report herein a case of T-ALCL, with an advanced stage, pulmonary involvement, high HIV viral load, and low CD4 level. Anaplastic lymphoma kinase (ALK) protein expression was negative. Anthracyclin-based chemotherapy was unsuccessful. The literature review was performed focusing on incidence, clinical characteristics, prognosis, and physiopathology of ALCL in HIV patients. More data are needed to improve the knowledge of such cases and to define a better treatment approach.

## 1. Introduction

In the era of modern highly active antiretroviral therapy (HAART), incidence of non-Hodgkin's lymphoma (NHL) occurring in HIV patients has dramatically decreased, and even this incidence remains higher than in the general population [1–3]. NHL occurring in HIV patients has generally aggressive features. Rare cases of low-grade NHL have been described [4–6]. Histological subtypes commonly seen display a B-cell origin such as Burkitt, centroblastic, plasmablastic, or primary effusion lymphoma [1]. Nevertheless, several cases of T-cell NHL have been described [1, 7]. We report herein a case of anaplastic large-cell lymphoma (ALCL) of T phenotype.

## 2. Case Report

 A 33-year-old man was admitted in our hospital for fever. His past medical history was unremarkable. Clinical exam was normal. Because of mild abdominal pain, an abdominal CT scan was performed that revealed an extrinsic obstruction of the right ureter by lymphadenopathy. Serologic test for HIV virus was positive. A biopsy of a lymph node was proposed to the patient, but he denied any exploration and leaved the hospital.

Two months later the patient was admitted in our unit. He was febrile, and his general condition has dramatically worsened. Any peripheral lymphadenopathy was detected. A new CT scan revealed a great increase of abdominal lymph nodes and the appearance of bilateral pulmonary nodules. CD4 cells count was 36/mm^3^, and HIV viral load was 366 000 copies/mL. LDH was 5 times above normal values. A bronchial biopsy was performed that revealed a massive invasion by median and large histiocyte-like cells with a high mitotic index. In places, some appearances of erythrophagocytosis were seen. Between these histiocytic cells, several atypical lymphoid cells were seen. Immunohistochemical staining of these lymphoid cells showed a positivity for CD2, CD4, CD30, and EMA and a negativity for CD3, CD7, CD8, and EBER-1. Cytogenetic analysis was not performed, but anaplastic lymphoma kinase (ALK) protein expression was negative. The diagnosis of lymphohistiocytic variant of T-ALCL ALK-negative was performed. Despite chemotherapy (CHOP regimen, i.e., cyclophosphamide, doxorubicin, vincristine, and prednisone), the patient's condition worsened, and he died after few days from multiorgan failure secondary to a severe macrophage activation syndrome.

## 3. Discussion

NHLs occurring in the context of HIV disease are mostly of B-cell origin. Nevertheless, T-cell NHL has been described, and several data argued in favour of an increased incidence of T-cell lymphoma in HIV patients. In a cohort of 6788 HIV NHL, 1.4% had T-cell lymphomas [7] with a relative risk of 15 above the rate observed in the general population. Another cohort of 429 AIDS-related lymphomas reported 11 cases (2.6%) of T cell origin [8]. T ALCL is a rare event in the context of HIV infection. Approximately 50 cases have been reported so far [9–11]. The proportion of ALCL in HIV patients with T-cell lymphoma was 22% in a series of 51 patients reported by Castillo et al. [12], 28% in a review of 85 patients reported by Castillo et al. [13], and 27% in a small series of 11 patients reported by Arzoo et al. [8].

ALCL in HIV-negative and -positive patients shares several common clinical features. An advanced disease (stage III or IV) was observed in the great majority of patients [9, 10]. Extranodal involvement is common. As for our patient, pulmonary involvement is present in 25% of cases.

In contrast, molecular signature of ALCL, that is, *t*(2; 5)(p23; q35), seems to be more frequent in HIV-negative ALCL (about half of the patients reported [14]) and low in HIV-positive ALCL (0–11%) [9, 12]. This translocation induces a fusion between the nucleophosmin (NPM) gene on chromosome 5q35 to ALK gene on 2p23 leading to the activation of ALK. Other translocations implicating ALK have been described such as *t*(1; 2)(q21; p23), *t*(2; 3)(p23; q21), and *t*(2; 17)(p23; q23). ALK is then fused to another gene partner than NPM [14]. 

Regarding the prognosis of ALK expression, conflicting data have been reported in HIV-negative ALCL patients. ALK expression is generally considered as a favourable criterion. In a recent series of 138 HIV-negative ALCL patients, any prognostic impact of ALK expression was seen in multivariate analysis [15]. The apparent poor prognosis of ALK-negative was explained by the more aggressive features and more advanced International Prognosis Index [15]. Prognosis of HIV-positive ALCL is generally very poor. In a review of 32 and 24 published cases, median survival was only 5 months and 6 months, respectively [9, 13]. In the review by Castillo et al., a majority of these patients received a CHOP regimen. The most common causes of death were lymphoma progression (37%) and opportunistic infections (31%) [9].

Data on physiopathology of HIV-positive ALCL are very poor. As we mentioned before, rearrangement of ALK gene seems to be very rarely implicated. HHV-8 has not been associated with HIV ALCL [9]. EBV was detected only in a minority of cases.

In conclusion, ALCL in HIV patients is a rare event. Prognosis is often very poor with standard treatments. So, alternative approaches must probably be used. Intensive chemotherapy with autologous stem cell transplantation could be an interesting approach, since a recent survey published by the EBMT has demonstrated identical results for HIV-positive patients and HIV-negative patients with this procedure [16, 17]. The experimental anti-CD30 antibody-drug conjugate (brentuximab vedotin) has given promising results in HIV-negative ALCL [18] but, to our knowledge, has not been tested in HIV patients.
